# Identification of lncRNA and mRNA Biomarkers in Osteoarthritic Degenerative Meniscus by Weighted Gene Coexpression Network and Competing Endogenous RNA Network Analysis

**DOI:** 10.1155/2020/2123787

**Published:** 2020-05-20

**Authors:** Jun Zhao, Yu Su, Jianfei Jiao, Zhengchun Wang, Xiangchun Fang, Xuefeng He, Xiaofeng Zhang, Zhao Liu, Xilin Xu

**Affiliations:** ^1^Department of Orthopaedics, Heilongjiang University of Chinese Medicine, Heping Road, Xiangfang District, Harbin, Heilongjiang, China; ^2^Harbin Fifth Hospital, Jiankang Road, Xiangfang District, Harbin, Heilongjiang, China

## Abstract

**Background:**

Long noncoding RNAs (lncRNAs) play a crucial role in varieties of biological processes. This study is aimed at investigating meniscal degeneration-specific lncRNAs and mRNAs and their related networks in knee osteoarthritis (KOA).

**Methods:**

The dataset GSE98918, which included 24 meniscus samples and related clinical data, was downloaded from the Gene Expression Omnibus database. The differentially expressed lncRNAs and mRNAs in the meniscus between KOA and control groups were identified. Based on the enriched differentially expressed lncRNAs and mRNAs, we constructed the coexpression network using WGCNA (weighted correlation network analysis) and identified the critical module related to KOA. For mRNAs in the key module, gene ontology and Kyoto Encyclopedia of Genes and Genomes pathway enrichment analyses were carried out using the DAVID database. A competing endogenous RNA network (ceRNA) based on the screened mRNAs, lncRNAs, and related miRNAs was constructed to reveal presumptive biomarkers further. Finally, the hub lncRNAs and mRNAs were screened, and the diagnostic value was evaluated using a receiver operating characteristic (ROC) curve. Hub mRNAs were validated using the dataset GSE113825.

**Results:**

We screened 208 significantly differentially expressed lncRNAs and mRNAs in menisci between the KOA and non-KOA samples, which were enriched in sixteen modules using WGCNA, especially the green module. Coexpression network based on the enriched differentially expressed lncRNAs and mRNAs in the green module uncovered 5 lncRNAs and 56 mRNAs. The lncRNA-miRNA-mRNA ceRNA network revealed that lnc-HLA-DQA1-5, lnc-RP11-127H5.1.1-1, lnc-RTN2-1, IGFBP4 (insulin-like growth factor binding protein 4), and KLF2 (Kruppel-like factor 2) were significantly correlated with the meniscus degeneration of KOA. ROC curve analysis revealed that these hub lncRNAs and mRNAs showed excellent diagnostic value for KOA.

**Conclusions:**

These hub lncRNAs and mRNAs were potential prognostic biomarkers for the meniscus degeneration of KOA. Further studies are required to validate these new biomarkers and better understand the pathological process of the meniscus degeneration of KOA.

## 1. Introduction

OA is one of the prevalent causes of worldwide disability [1], and its incidence is climbing owing to an aging population and increasing obesity [2]; moreover, KOA accounts for more than 80% [3]. The epidemiological investigation showed that 14 million persons had symptomatic KOA, of which nearly 2 million were younger than 45 years old, and over half were advanced KOA in America [4]. Individuals with KOA usually suffer from pain and stiffness that eventually lead to associated functional loss. However, current palliative care can alleviate short-term symptoms in early-stage KOA, but in the long run, degeneration and damage of meniscus seem inevitable [5]. Total knee replacement (TKR), which is proven to be effective but very expensive, is considered to be the ultimate treatment for end-stage KOA [4, 6]. What is terrible is that late mortality is higher than expected after knee arthroplasty [6]. Therefore, it seems urgent to delay the progression of KOA by early diagnosis and treatment.

During the progression of OA, the functional properties and structure of the articular cartilage undergo gradual changes [7], such as degenerative loss of articular cartilage, subchondral bone sclerosis, and synovial inflammation [8]. As an internal structure, the meniscus is essential for healthy knee joints and plays a critical role in the progression of KOA [9]. Knee menisci undergo many histopathological changes in the progress of KOA, such as tears, calcification, and atypical cell arrangement [10]. Meniscal damages can contribute to KOA; in turn, KOA can also give rise to meniscal tears through structural destruction and degeneration of the meniscus [11]. Moreover, substantial evidence indicated that 60% of asymptomatic KOA patients had MRI-verified meniscal tears [12]. Many studies have focused on gene expression profiles of subchondral bone, or synovium in patients with KOA [13–15], but few consensus biomarkers for the meniscus, especially lncRNAs. Although lncRNAs do not encode proteins, it plays a critical functional role in the whole transcriptional process [16]. For instance, a study showed that lnc-HOTAIR and FUT2 were upregulated in OA cartilage, and overexpressed lnc-HOTAIR indirectly regulated the expression of FUT2 through sponging miR-17-5p to aggravate chondrocyte apoptosis and injury, which may make lncRNA a new molecular therapeutic target for OA [17].

In this study, to explore the role of lncRNAs and mRNAs in patients with KOA, we extracted a meniscal degeneration-associated lncRNA-mRNA network from WGCNA [18]. Then, a lncRNA-miRNA-mRNA ceRNA network based on the screened mRNAs, lncRNAs, and related miRNAs was constructed to reveal presumptive biomarkers further. Additionally, the potentials of susceptibility lncRNAs and mRNAs for the prediction of KOA were also investigated based on the ROC curves. Therefore, microarray analysis of meniscus samples may be beneficial to identify new biomarkers and increase the necessary mechanical understanding for early prevention of KOA.

## 2. Materials and Methods

### 2.1. Data Collection

GSE98918 (deposited by Brophy et al. [19]) and GSE113825 (stored by Zhang et al. [20]) were downloaded from the Gene Expression Omnibus (GEO) database (https://www.ncbi.nlm.nih.gov/geo/). GSE98918 included 24 meniscus samples, 12 KOA and 12 non-KOA samples, and related clinical data, and the platform is Agilent-072363 SurePrint G3 Human. As for the validated dataset GSE113825, it contains five cartilage-OA samples and five cartilage-normal samples, and its platform is Affymetrix Human OElncRNAs520855F Array.

### 2.2. Data Preprocessing and Screening of Differentially Expressed lncRNAs and mRNAs

According to the annotation information of the GPL20844 platform, the data probes were transformed to gene symbols. When genes correspond to multiprobes, the average value of its expression was calculated. Then, the KNN method of the impute package of R was used to add the missing values of probes, and quantile normalization was carried out [21]. Finally, gene symbols were reannotated into a complete human genome (GRCh38), and the expression levels of lncRNAs and protein-coding mRNAs were obtained. Differentially expressed lncRNAs and mRNAs were identified by limma package [22] in R (version 3.6.1) with the cut-off criteria of *P* value of <0.05 after adjustment, and ∣log2FC | >1.

### 2.3. Gene Ontology (GO) and KEGG (Kyoto Encyclopedia of Genes and Genomes) Pathway Enrichment Analyses

GO enrichment analysis including biological process, cellular component, and molecular function categories and KEGG pathway enrichment analysis were used to further clarify the potential mechanism of differentially expressed mRNAs by DAVID 6.8 database (https://david.ncifcrf.gov/home.jsp) [23]. *P* value < 0.05 was selected as the threshold.

### 2.4. WGCNA Analysis to Screen-Specific Modules and RNAs

WGCNA can be used to construct a weighted gene coexpression network, find coexpression modules, and screen candidate targets for diseases. In this study, the modules were detected using the dynamic tree cut algorithm with a minimum module size of 30. Combined with clinical data, the coexpression modules of lncRNAs and mRNAs, which were mostly related to meniscus degeneration in patients with KOA, were obtained. Differentially expressed RNAs were mapped to the critical modules screened by WGCNA.

### 2.5. Construction of lncRNA and mRNA Coexpression Network

The cor function in R language was used to calculate the Pearson correlation coefficient (PCC) of differentially expressed lncRNAs and mRNAs in the critical modules of WGCNA. The cut-off of PCC was 0.6. Then, the coexpression network was constructed and visualized using Cytoscape 3.7.1 [24]. Finally, we performed the GO and KEGG pathway enrichment analyses of those mRNAs.

### 2.6. ceRNA Network Construction

According to the theory that lncRNAs can affect miRNAs and further regulate mRNA expression as a miRNA sponge, we constructed a ceRNA network. First, the targeted miRNAs for enriched differentially expressed lncRNAs in the green module were identified through the lncRNASNP2 database (http://bioinfo.life.hust.edu.cn/lncRNASNP2) [25], and the lncRNA-miRNA relation pairs were constructed. Second, TargetScan (http://www.targetscan.org/), miRTarBase (http://mirtarbase.mbc.nctu.edu.tw/), and miRDB (http://www.mirdb.org/) [26] were used to predict target genes of the miRNAs, in which mRNAs that overlap with the differential expression of the green module were used to construct miRNA-mRNA interaction pairs. Finally, the lncRNA-miRNA-mRNA ceRNA network was established and visualized using Cytoscape 3.7.1.

### 2.7. Statistical Analysis

Statistical analysis was performed using GraphPad Prism 8.0 (GraphPad Software, Inc.). One-way analysis of variance or the nonparametric Wilcox test was used to evaluate significant differences between the patients with KOA and the control samples. ROC curves were utilized to assess the biomarker accuracy. *P* value < 0.05 was chosen as the cut-off criteria.

## 3. Results

### 3.1. Identification of Differentially Expressed mRNAs and lncRNAs

**Figure 1:**
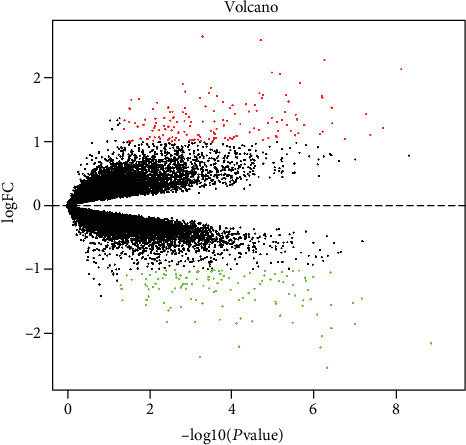
Volcano plot of the aberrantly expressed lncRNAs and mRNAs in the meniscus between KOA and control groups. Red: high expression; green: low expression; black dots: represent lncRNAs and mRNAs, which are not differentially expressed.

A total of 18428 protein-coding genes and 10988 lncRNAs were obtained after reannotation. Based on the cut-off criteria, 32 lncRNAs (including 21 upregulated and 11 downregulated) and 176 mRNAs (including 74 upregulated and 102 downregulated) were significantly differentially expressed (Table S1), as shown in Figure 1.

### 3.2. Functional Enrichment Analysis of the Differentially Expressed mRNAs

**Figure 2:**
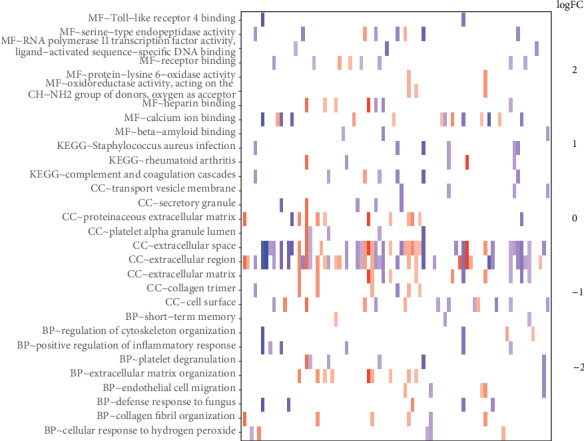
GO and KEGG pathway enrichment analyses of differentially expressed mRNAs. The heat map of KEGG results and 9 most significantly GO results. Red: upregulated mRNAs; blue: downregulated mRNAs; MF: molecular function; KEGG: Kyoto Encyclopedia of Genes and Genomes; CC: cellular component; BP: biological processes.

To further investigate the function of differentially expressed mRNAs, respective GO and KEGG analyses were conducted using the DAVID online database. According to the *P* value (*P* < 0.05) of each term (Table S2), as shown in Figure 2, these differentially expressed protein-coding RNAs associated with GO functions of collagen fibril organization, extracellular matrix organization, and endothelial cell migration, as well as KEGG pathways of Staphylococcus aureus infection (hsa05150), complement and coagulation cascades (hsa04610), and rheumatoid arthritis (hsa05323).

### 3.3. Identification and Characterization of KOA-Associated Modules Using WGCNA

**Figure 3:**
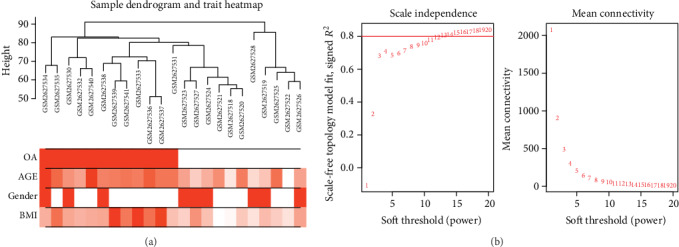
WGCNA processing for GSE98918. (a) Sample dendrogram and trait heat map, the color band underneath the tree represents stages which indicate KOA and non-KOA, age, gender, and BMI (red indicates high values); (b) power value for the adjacency matrix in WGCNA, where the red line signals 0.8 on the vertical axis. *β* = 14 was chosen for subsequent analysis. OA: osteoarthritis; BMI: body mass index.

**Figure 4:**
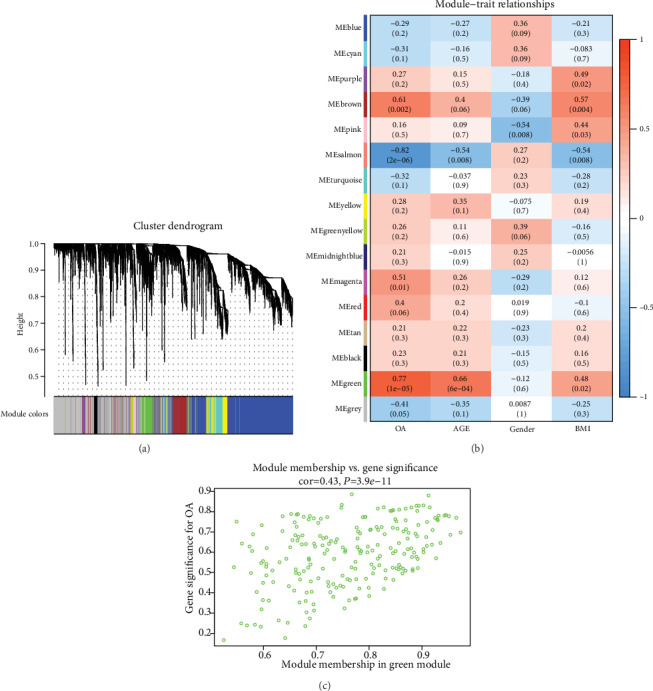
Screening of key modules related to KOA. (a) The cluster dendrogram of RNAs with each branch representing one RNA, and each color representative of a coexpression module. (b) Module-trait relationships, each row corresponds to a module and the column corresponds to different clinical features. The numbers in the cell represent corresponding *P* value and the correlation coefficient. (c) Scatterplot of gene significance for OA vs. module membership in the green module, one dot represents one RNA.

To explore the coexpression patterns of the meniscal degeneration-specific mRNAs and lncRNAs in KOA, WGCNA was used to construct the gene coexpression network. Based on variance analysis, the top 25% of RNAs (7353 RNAs) were obtained from GSE98918 with 24 samples. Sample dendrogram analysis showed the GSM2627529 sample was outlying, so it was excluded (Figure 3(a)). In the current study, the power of *β* = 14 (scale free *R*^2^ = 0.80) was set as the soft threshold for a scale-free network (Figure 3(b)). Sixteen coexpression modules were defined through the dynamic tree cutting method (Figure 4(a)). After relating those modules to clinical traits, correlations were observed in the trait of OA stage and the green module (*r* = 0.77, *P* = 1*e* − 05), including 202 protein-coding RNAs and 12 lncRNAs (Table S3), which had the highest correlation. Interestingly, modules that were highly related to the trait of age and weight were detected, such as the green module (*r* = 0.66, *P* = 6*e* − 04) and the brown module (*r* = 0.57, *P* = 0.004). However, there was no significant gender-related module, which was inconsistent with the related research [27] (Figure 4(b)). Moreover, based on an intramodular analysis, RNAs in the green module (cor = 0.43, *P* = 3.9*e* − 11, Figure 4(c)) were characterized by high module membership and gene significance. Differentially expressed RNAs in the green module included 5 lncRNAs and 56 mRNAs (Table S4).

### 3.4. Differential lncRNA-mRNA Coexpression Network Analysis Associated with KOA

**Figure 5:**
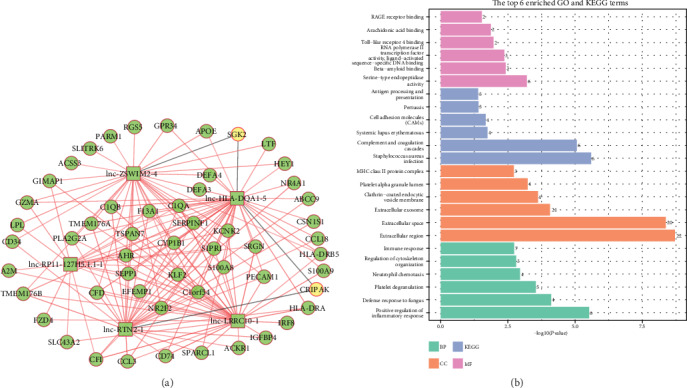
lncRNA-mRNA coexpression network. (a) Squares indicate lncRNA, and circles indicate mRNA. Green: upregulated RNAs; yellow: downregulated RNAs; grey lines represent negative correlations, and red lines represent positive correlations. (b) The top 6 enriched GO functions and KEGG pathways of differentially expressed mRNAs in the green module. BP: biological processes; CC: cellular component; KEGG: Kyoto Encyclopedia of Genes and Genomes; MF: molecular function.

To further explore the coexpression pattern and function of 5 differently expressed lncRNAs and 56 differently expressed mRNAs in the green module, we calculated the PCC of those lncRNAs and mRNAs (Table S5) and constructed the lncRNA-mRNA coexpression network which contained 59 nodes and 160 edges (156 positive and 4 negative connections), as shown in Figure 5(a). Among these RNAs, five lncRNAs were upregulated, while 52 mRNAs were upregulated, and two mRNAs were downregulated. Then, GO and KEGG enrichment analyses of mRNAs in the coexpression network were performed using the DAVID online database. The results indicated 8 KEGG pathways and 70 GO functions (44 biological processes, 7 molecular functions, and 19 cellular compartments) were enriched (Table S6 and Figure 5(b)), such as Staphylococcus aureus infection (hsa05150), complement and coagulation cascades (hsa04610), cell adhesion molecules (hsa04514), positive regulation of inflammatory, defense response to fungus, platelet degranulation, and serine-type endopeptidase activity.

### 3.5. ceRNA Network Further Revealed the lncRNA and mRNA Biomarkers Related to KOA

**Figure 6:**
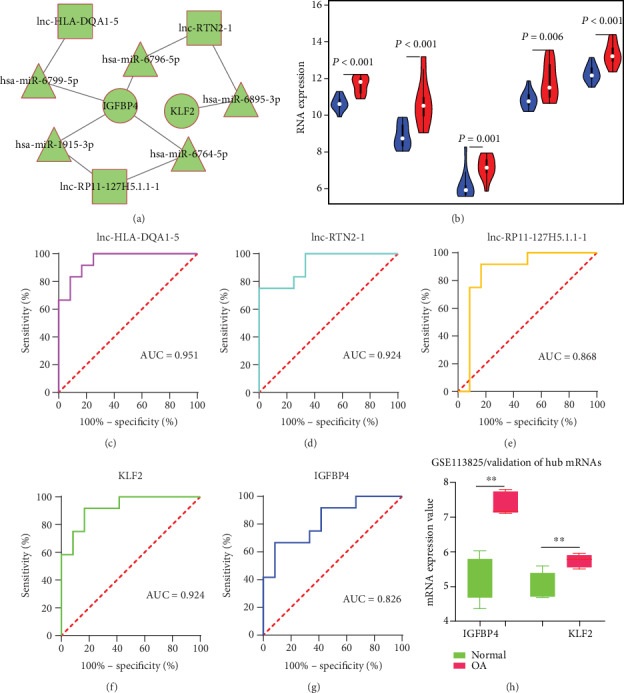
ceRNA network and hub RNA efficacy evaluation. (a) In network, gray edges indicate lncRNA-miRNA-mRNA interactions. Squares represent lncRNAs, triangles represent miRNAs, and circles represent protein-coding mRNAs. (b) The expression of five hub RNAs in GSE98918, red represents KOA samples and blue represents controls. (c–g) ROC curve of five hub RNAs for patients with KOA. AUC: the area under the ROC curve. (h) Validation of hub mRNAs in GSE113825. IGFBP4: insulin-like growth factor binding protein 4; KLF2: Kruppel-like factor 2; OA: osteoarthritis; ^∗∗^ indicates *P* < 0.01.

Based on the list of 5 differently expressed lncRNAs and 54 differently expressed mRNAs in the green module, we constructed the ceRNA network. First, the target predictions of lncRNA-miRNA pairs were retrieved from the lncRNASNP2 database, and 212 miRNAs were predicted as potential targets of 5 lncRNAs. Second, miRNA targeted mRNAs were obtained from the TargetScan, miRDB, and miRTarBase databases, and a total of 5 miRNA-mRNA pairs were identified, including 5 miRNAs and 2 mRNAs (Table S7). Finally, the lncRNA-miRNA-mRNA ceRNA network was constructed and visualized using Cytoscape 3.7.1, as shown in Figure 6(a). lncRNAs of lnc-HLA-DQA1-5, lnc-RP11-127H5.1.1-1, and lnc-RTN2-1 positively related to the mRNAs of IGFBP4 and KLF2 were potential biomarkers associated with KOA.

### 3.6. Hub RNA Efficacy Evaluation

In dataset GSE98918, the expression of five interested RNAs including lnc-HLA-DQA1-5, lnc-RP11-127H5.1.1-1, lnc-RTN2-1, IGFBP4, and KLF2 was significantly increased in the degenerative meniscus of KOA (Figure 6(b)), highlighting the notable role of these RNAs in the osteoarthritic degenerative meniscus. Besides, the area under the curve (AUC) was calculated to evaluate the significance. As depicted in Figures 6(c)–6(g), every AUC of the five involved RNAs was >0.8, highlighting the ability of these five interested RNAs as potential markers for KOA, especially lnc-HLA-DQA1-5, lnc-RTN2-1, and KLF2 (AUC > 0.9). Finally, the result of the validation of IGFBP4 and KLF2 from the dataset GSE113825 is displayed in Figure 6(h).

## 4. Discussion

OA is a common degenerative disease of joints, and the degeneration of articular cartilage is the primary pathological mechanism [28, 29]. Preventing the degeneration of articular cartilage, especially the meniscus, is crucial to the early treatment of KOA [29]. Therefore, understanding the potential pathogenesis of meniscus degeneration is of considerable significance to identify the therapeutic targets and is beneficial to study the disease further and to search for the biotherapies of the disease. Accumulating studies highlighted the role of lncRNAs in disease pathology from the view of gene expression regulation [21, 30, 31]. To illuminate the mechanism of meniscus degeneration of KOA, we used differential expression analysis, WGCNA, and ceRNA networks to identify biomarkers in samples of the meniscus from KOA and non-KOA subjects.

In the present study, 208 differentially expressed RNAs, including 32 lncRNAs and 176 mRNAs, that were primarily involved in collagen fibril organization, extracellular matrix organization, endothelial cell migration, Staphylococcus aureus infection, complement and coagulation cascades, and rheumatoid arthritis were obtained. Moreover, the green module, including 202 mRNAs and 12 lncRNAs, was associated with the occurrence of KOA by WGCNA analysis. Based on the analysis results, we calculated the PCC of the green module differentially expressed 5 lncRNAs and 56 mRNAs to construct the lncRNA and mRNA coexpression network, which contained 156 positive and 4 negative connections. The green module with 56 differentially expressed mRNAs played an essential role in positive regulation of inflammatory response, platelet degranulation, Staphylococcus aureus infection, cell adhesion molecules, etc. Then, using miRNA as a bridge, the ceRNA network was constructed by screening the paired lncRNA-miRNA-mRNA. Finally, the upregulated lnc-HLA-DQA1-5, lnc-RP11-127H5.1.1-1, lnc-RTN2-1, IGFBP4, and KLF2 were identified as the hub RNAs, and every AUC of the ROC curve of the 5 RNAs supported the analysis result. Besides, IGFBP4 and KLF2 demonstrated significant results in the GSE113825 validation.

IGFBP4, a member of the IGFBP family, has a high affinity with IGF (insulin-like growth factor) [32] and is an effective IGF inhibitor [33], while IGF is essential for the differentiation, development, and maintenance of bone tissue [34]. Proteolysis of IGFBP4 can increase the bioavailability of IGF, resulting in a significant increase in bone formation parameters in mice [33]. Fu et al. reported that IGFBP4 expression in the articular cartilage of OA patients was significantly upregulated [35], which was consistent with the results of our analysis. Besides, overexpression of IGF-I in OA chondrocytes could dramatically reduce the expression of IGFBP4 and stimulate cellular activities [36], which provided a driving force for the future study of the mechanism of meniscal degeneration of KOA. KLFs, a group of transcription factors, are a vital part of the eukaryotic transcription mechanism in cells [37]. KLF2 is a member of the KLF family that not only regulates immune cell function but also plays a critical regulatory role in certain abnormal and pathological conditions, such as inflammation, adipose differentiation, and autophagy [38, 39]. Contrary to our results, Gao et al. reported that the expression of KLF2 was significantly decreased in the cartilage of KOA [40], which may be due to the selection of tissue samples and differences in KOA grades because the expression pattern of specific genes depends on the stage of KOA [41]. Besides, KLF2 can regulate the expression of MMP-13 in different ways to prevent OA [40, 42]. Therefore, we infer that the dynamic changes of KLF2 expression in meniscus tissues at various stages of KOA may be one of the potential therapeutic targets of KOA.

It has been demonstrated that lncRNAs have diverse functions such as regulation of RNA or protein molecules, transcriptional regulation cis or trans, and coding small proteins [43]. Chen et al. [44] showed that the expression level of lnc-RTN2-1 (AC138128.1) in gastric cancer was significantly lower than that in paracancer tissues and associated with the clinicopathological features of gastric cancer. Also, Xiao et al. [45] reported that the lnc-RTN2-1 expression in lung cancer tissue was decreased significantly compared to that in paracancer tissues, and lnc-RTN2-1 increased in a dose- or time-dependent manner after in vitro anticancer drug treatment. These suggested that lnc-RTN2-1 might be of potential value in predicting the diagnosis and treatment of diseases. Qian et al. [46] found that the lnc-HLA-DQA1-5 (ENST00000419852, HLA-DQB1-AS1) expression in patients with smoking chronic obstructive pulmonary disease (COPD) was increased compared to controls and speculated that it played a crucial role in the pathogenesis in smoking COPD. Admittedly, accumulating evidence supported that there were many differentially expressed lncRNAs in KOA cartilage [35, 47] and that lncRNAs were involved in the formation, proliferation, apoptosis, and autophagy of chondrocytes [47, 48]. For example, FOXD2-AS1 induced the proliferation of chondrocytes in OA by sponging miR-27a-3p [49], lncRNA-CIR regulated the apoptosis of chondrocytes in OA [50], and overexpression of lncRNA-ROR significantly promoted the viability of OA chondrocytes and regulated the autophagy and apoptosis of chondrocytes through p53 and HIF1*α* [51]. Tian et al. [52] verified that lncRNA SNHG7 expression was downregulated in OA cartilage tissues compared with healthy cartilage tissues and overexpressed lncRNA SNHG7 inhibited cell apoptosis and autophagy and promoted cell proliferation by regulating the miR-34a-5p/SYVN1 axis. However, there were few studies on the mechanism of lncRNAs involved in cartilage or meniscus degeneration in KOA, such as lnc-HLA-DQA1-5, lnc-RP11-127H5.1.1-1, and lnc-RTN2-1. In this study, based on the analysis of WGCNA and ceRNA network, we found that lnc-HLA-DQA1-5, lnc-RP11-127H5.1.1-1, and lnc-RTN2-1 participated in the mechanism of meniscus degeneration by regulating the expression of IGFBP4 and KLF2 through sponging miRNAs (miR-6799-5p, miR-1915-3p, miR-6764-5p, miR-6796-5p, and miR-6895-3p), which were related to the pathways of Staphylococcus aureus infection, complement and coagulation cascades, and cell adhesion molecules. But the functional verification of the experiment has not been carried out. Nevertheless, this work provides novel insights into the occurrence and development of meniscus degeneration in KOA.

## Data Availability

The data used to support the findings of this study are included within the article.

## Supplementary Materials

Supplementary materials Table S1: significantly differentially expressed RNAs. Table S2: the results of GO and KEGG analyses of differentially expressed mRNAs. Table S3: lncRNAs and mRNAs in the green module. Table S4: differentially expressed lncRNAs and mRNAs in the green module. Table S5: the PCC of differentially expressed lncRNAs and mRNAs in the green module (the cut-off was PCC > 0.6 or PCC < −0.6). Table S6: the results of GO and KEGG analyses of screened mRNAs. Table S7: the results of lncRNA-miRNA-mRNA network.
